# Behaviorally Informed Text Messaging to Promote Colon Cancer Screening: A Quality Improvement Randomized Clinical Trial

**DOI:** 10.1001/jamanetworkopen.2026.7122

**Published:** 2026-04-23

**Authors:** Olivia Korostoff-Larsson, William C. King, Elan Pelegri, Doreen Colella, Isaac Dapkins, Kelly Eng, Nathan Klapheke, Holly Krelle, Nicholas Mahieu, Erika McManus, George Shahin, Molly Woodriff, Leora I. Horwitz, Arielle Elmaleh-Sachs

**Affiliations:** 1 Division of Healthcare Delivery Science, Department of Population Health, NYU Grossman School of Medicine, New York, New York; 2 Family Health Centers at NYU Langone, New York, New York; 3 Department of Medicine, NYU Grossman School of Medicine, New York, New York; 4 Medical Center Information Technology, NYU Langone Health, New York, New York

## Abstract

**Importance:**

Colorectal cancer screening rates in the US remain suboptimal, particularly among low-income and minoritized populations, despite the availability of effective, low-cost options such as the fecal immunochemical test (FIT). Scalable outreach strategies are needed to improve uptake and reduce staff burden in safety-net settings.

**Objective:**

To evaluate whether a behavioral economics–informed, automated text messaging strategy was associated with increased FIT completion compared with nurse-led telephone call outreach.

**Design, Setting, and Participants:**

This quality improvement randomized clinical trial was conducted from April 7 to June 24, 2025, at 8 Federally Qualified Health Centers (FQHCs) in Brooklyn, New York, within the Family Health Centers at NYU Langone. Participants included adults (aged ≥18 years) with a new FIT order who listed English, Spanish, or Chinese (Mandarin or Cantonese) as their preferred language and had not opted out of text messaging.

**Intervention:**

Patients were randomized 1:1 to receive either 3 automated, 1-way text message reminders on days 2, 5, and 8 (intervention) or a single nurse-led telephone call reminder on day 8 (usual care).

**Main Outcomes and Measures:**

The primary outcome was FIT completion within 21 days of the test order, assessed from the electronic health record. Secondary outcomes included completion at 7 and 14 days. FIT completion at 7, 14, and 21 days was compared between groups using χ^2^ tests.

**Results:**

Among 1275 eligible randomized participants, 649 were assigned to the text group (418 female participants [64.4%]; mean [SD] age, 56.4 [9.3] years) and 626 to the telephone group (398 female participants [63.6%]; mean [SD] age, 56.7 [9.6] years). FIT completion within 21 days was higher in the text group (382 of 649 participants [58.9%]) compared with the telephone group (312 of 626 participants [49.8%]) with an absolute difference of 9.0 percentage points (95% CI, 3.6-14.5 percentage points; *P* = .001). Post hoc analyses found no evidence of differential effectiveness by age, sex, race and ethnicity, or patient portal use.

**Conclusions and Relevance:**

In this quality improvement randomized clinical trial, a behaviorally informed text messaging strategy was associated with significantly improved FIT completion compared with usual nurse-led telephone outreach. Automated messaging may offer a scalable, low-cost strategy to promote preventive care and reduce staff burden in underserved populations.

**Trial Registration:**

ClinicalTrials.gov Identifier: NCT06632054

## Key Points

**Question:**

Is a behavioral economics–informed, automated text messaging strategy associated with improved colorectal cancer screening uptake compared with usual nurse-led telephone call outreach in a safety-net population?

**Findings:**

In this quality improvement randomized clinical trial of 1275 adults with new fecal immunochemical test (FIT) orders, 58.9% of participants in the text message group completed screening within 21 days compared with 49.2% in the telephone call group, a statistically significant difference.

**Meaning:**

These findings suggest that automated, behaviorally informed text messages were associated with significantly improved FIT completion compared with nurse-led telephone outreach and may provide a scalable, low-cost strategy to promote preventive care in underserved populations.

## Teaser

This quality improvement randomized clinical trial evaluates whether a behavioral economics–informed, automated text messaging strategy is associated with increased fecal immunochemical test completion compared with nurse-led telephone call outreach.

## Introduction

Colorectal cancer (CRC) screening is a proven strategy for early detection and mortality reduction, yet uptake remains suboptimal across the US.^1^ As of 2023, only 72.6% of adults aged 50 to 75 years were up to date with screening, falling short of national targets.^2^ Screening rates are even lower among historically underserved populations. At Federally Qualified Health Centers (FQHCs), just 41.2% of patients were up to date with CRC screening as of 2022.^2^ Individuals from low-income and minoritized groups are less likely to undergo timely screening^3,4,5^ and more likely to receive a diagnosis of advanced-stage disease.^6^ At the Family Health Centers at NYU Langone (FHC), an FQHC network in Brooklyn, New York, 59% of eligible adults were up to date with screening in 2024, higher than the FQHC average, but still well below national targets. These gaps underscore the need for scalable interventions to improve screening uptake in underserved populations.

Among available CRC screening modalities, the fecal immunochemical test (FIT) is a widely used, stool-based method recommended annually for average-risk adults.^7,8^ FIT is effective at reducing cancer incidence and mortality, and its low cost and ease of distribution have led many organized CRC screening programs to adopt it as their primary screening modality.^9,10,11^ Despite its advantages, FIT completion remains a challenge, often because of logistical or cognitive barriers, such as forgetting to complete the test, uncertainty about instructions, or discomfort around handling stool.^12^ In response, a growing body of research has explored ways to increase FIT return rates. Interventions such as mailed FIT outreach and patient navigation have demonstrated success across diverse populations,^13^ but these approaches can be resource-intensive, limiting their feasibility and scalability in safety-net settings.

To improve screening uptake, some programs have adopted low-cost strategies grounded in behavioral economics. These strategies, often referred to as *nudges*, work by subtly reshaping how choices are presented to make the desired action, such as returning a FIT kit, more intuitive, urgent, or socially desirable.^14^ Effective examples from prior studies include notifying patients in advance of mailing a FIT kit (to support planning and preparation),^15^ opting patients into screening by default (to reduce decision inertia),^16^ and sending reminders signed by a trusted practitioner (providing social pressure).^15,16,17,18,19,20,21^ Adding a return-by deadline to screening reminders has also been shown to increase FIT completion in randomized studies.^20,21^ Nudges such as these can be easily embedded within reminder messages, with text messaging in particular offering a low-cost, scalable delivery method that is adaptable to local workflows and easy to refine over time. In prior work within our FQHC network, for example, we found that small adjustments to the framing and timing of text messages were associated with significantly improved vaccination uptake.^22^

Building on prior evidence, we conducted a randomized test of a behavioral economics–informed text message reminder strategy designed to increase FIT completion at a large FQHC network. The intervention and randomization were embedded in routine clinical care. We hypothesized that this approach would increase FIT return rates compared with standard telephone outreach and offer a scalable model for improving uptake of preventive care.

## Methods

We conducted a quality improvement randomized clinical trial at the FHC, a network of 8 FQHC sites in Brooklyn, New York, serving over 100 000 patients annually. This study was determined to be a quality improvement initiative by the NYU Langone Health Quality Improvement Oversight Committee and was not formally supervised by the institutional review board per NYU Langone Health institutional policies. Participant consent was not required. We followed the Standards for Quality Improvement Reporting Excellence (SQUIRE) reporting guidelines.^23^

Enrollment occurred from April 7 to June 24, 2025, with follow-up through July 15, 2025, to allow 21 days for FIT completion. The primary objective was to evaluate whether a behaviorally informed text message reminder could increase FIT completion compared with standard telephone outreach. We hypothesized that automated outreach incorporating behavioral nudges would improve screening uptake. The trial protocol is shown in Supplement 1.

### Participants

Eligible participants were adults (aged ≥18 years) with a new FIT order placed between April 7 and June 24, 2025, who had not opted out of text messaging and listed English, Spanish, or Chinese (Mandarin or Cantonese) as their preferred language. Patients preferring other languages or who opted out of texts were excluded and continued to receive usual care. Patients with FIT orders who were part of the FHC’s Community Medicine Program were also excluded from randomization because that program had an ongoing CRC screening study. Finally, patients who had completed a FIT within the prior 12 months were excluded because, per clinic protocol, they were not eligible to receive nurse-led reminder telephone calls. This was intended to reserve nursing time for patients in need of updated screening. Given technical limitations in identifying these patients in real time, those with recent FITs were manually excluded after randomization: they were either screened out by the callers in the telephone group (thereby not receiving calls) or were manually removed by the study team from the text group after being sent a message.

Eligible participants were randomized 1:1 to receive text message reminders (intervention) or standard nurse-led telephone outreach (control) using a computer-generated sequence at the time of FIT order. Because of the nature of the intervention, neither staff nor participants were blinded.

### Interventions

In both groups, practitioners were prompted to order annual FIT screening for adults aged 45 years and older. Practitioners could also order screening for younger patients at their discretion. FIT kits were distributed in person (or mailed after virtual visits) at no cost to the patient, with nurses providing instructions. In the control group, patients with unreturned kits received a single telephone call reminder from a nurse on day 8, per clinic protocol. Call completion varied across sites based on staffing and workload and was not modified for the study. When patients did not answer and voicemail was available, nurses left a message that explicitly reminded patients to return the FIT and included a callback number for questions. Intervention group patients received up to 3 automated text messages on days 2, 5, and 8. Messages were withheld if FIT completion was logged earlier. Each message included the patient’s clinic address and a centralized telephone number for questions or replacement kits. Message content was developed with clinic input and underwent review for health literacy and communication best practices. Messages were sent via NYU Langone Health’s existing text platform and in the patient’s preferred language (English, Spanish, or Chinese).

The messages incorporated several behavioral science elements (Table 1). To encourage timely completion, they included a specific due date (day 9 after the order), leveraging the motivating effect of deadlines.^20,21^ They also invoked social norms by referencing the patient’s practitioner in the second message (“your provider is waiting to receive…”), fostering a sense of accountability.^17,18,19^ The messages employed gain-framing by emphasizing that screening may “save your life,” highlighting the potential benefits of action. Finally, repetition was used by sending 3 reminders, reinforcing the importance of the task and mitigating forgetfulness.^24^

**Table 1.** Text Message Language Sent on Days 2, 5, and 8

Day sent after FIT order	Message language
2	“NYU Langone: Your provider gave you a colon cancer stool screening test at your recent appt. The test is easy to complete and can save your life! Please return it by [send_by_date] to [clinic_address].”
5	“NYU Langone: Your provider is waiting to receive your colon cancer stool screening test. If you have already returned your test, thank you! If you need a new test or need help, please call [central_number].”
8	“NYU Langone: Your colon cancer stool screening test is due tomorrow at [clinic_address]. If you have already returned it, thank you! For help, call the clinic at [central_number].”

Abbreviation: FIT, fecal immunochemical test.

### Outcomes

The primary outcome was FIT completion, defined as return and laboratory processing of the test kit within 21 days of the order, as recorded in the electronic health record (EHR). We also assessed completion at 7 and 14 days. Post hoc exploratory subgroup analyses evaluated potential differences in intervention effects by sex, age group, self-reported race and ethnicity (categorized as Asian; Black or African American; Hispanic, Latino, or Spanish; White; or other, defined as non-Hispanic American Indian or Alaska Native, Native Hawaiian or Other Pacific Islander, Middle Eastern or North African, multiracial, and any race or ethnicity not otherwise specified), preferred language, and patient portal engagement. All variables were extracted from the EHR. Data on race and ethnicity were included because of known differences in colorectal cancer screening rates by race and ethnicity.

### Sample Size

We estimated that enrolling 720 participants would provide 80% power to detect a 10–percentage point absolute difference in FIT completion between groups (2-sided α = .05). This threshold was selected as the minimum clinically meaningful difference. We decided to enroll twice as many participants to allow for additional analyses of differences in effect among population subgroups.

###  Statistical Analysis

Analyses followed an intention-to-treat approach among eligible patients. FIT completion at 7, 14, and 21 days was compared between groups using χ^2^ tests, performed in SAS statistical software 9.4 (SAS Institute). We also fit a logistic regression model with treatment assignment as the independent variable to estimate the overall intervention effect while controlling for race, ethnicity, age, insurance type, sex, and patient portal use. From this model, we calculated average marginal effect (AME) with a 95% CI using robust SEs.

To examine whether the intervention effect varied across patient subgroups, we then fit separate logistic regression models that included an interaction term between treatment assignment and each covariate of interest (race and ethnicity, age, insurance type, sex, and patient portal use). For each model, we evaluated the coefficient for the interaction term and its *P* value to assess evidence of heterogeneity relative to the reference category. All regression analyses were conducted in R statistical software version 4.4.1 (R Project for Statistical Computing). Statistical significance was defined as 2-sided *P* < .05.

To estimate the operational impact of replacing telephone calls with text messages, we calculated the average number of calls made per month in the call group and estimated that the calls on average, including completed and attempted calls, required 5 minutes of staff time. To estimate the monthly number of additional completed FITs, we applied the observed AME of text messaging to the average monthly number of new FIT orders across all FHC sites. Using EHR data on the proportion of abnormal FIT results and published estimates of the positive predictive value of a onetime FIT, we projected the number of abnormal tests and CRC cases that would be detected if text messages were used in place of telephone calls.

## Results

### Study Participants

**Figure 1.**
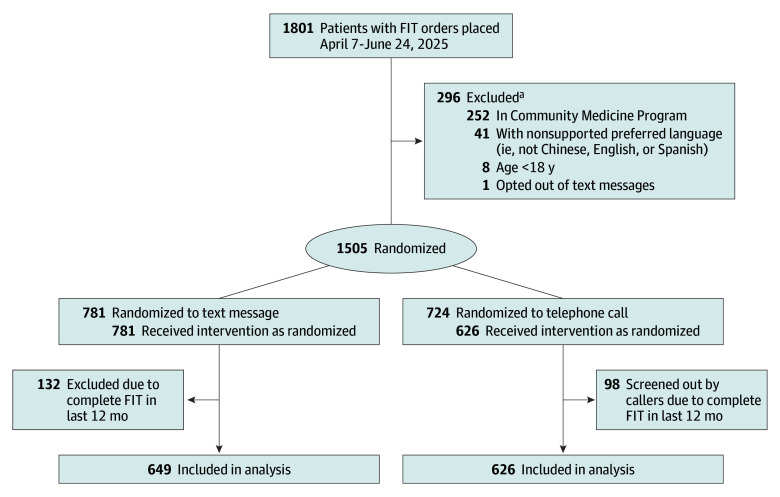
Flow Diagram of Participant Enrollment, Allocation, and Analysis FIT indicates fecal immunochemical test. ^a^Patients may have had multiple reasons for exclusion.

Between April 7 and June 24, 2025, 1275 eligible patients with new FIT orders were randomized: 649 to the text group and 626 to the call group (Figure 1). No one was lost to follow-up. Follow-up for the primary outcome concluded on July 15, 2025. Baseline characteristics were well-balanced between groups (Table 2). Participants were predominantly Hispanic (329 participants [50.7%] in text vs 317 participants [50.6%] in call), female (418 participants [64.4%] vs 398 participants [63.6%]), and Spanish-speaking (407 participants [62.7%] vs 372 participants [59.4%]). The mean (SD) age was also similar across groups (56.4 [9.3] vs 56.7 [9.6] years).

**Table 2.** Baseline Characteristics of Participants in Call and Text Groups

Variable	Participants, No. (%)	
	Call (n = 626)	Text (n = 649)
Sex		
Female	398 (63.6)	418 (64.4)
Male	228 (36.4)	231 (35.6)
Race and ethnicity		
Asian	80 (12.8)	70 (10.8)
Black or African American	60 (9.6)	55 (8.5)
Hispanic, Latino or Spanish	317 (50.6)	329 (50.7)
White	78 (12.5)	83 (12.8)
Other^a^	49 (7.8)	60 (9.2)
Prefer not to answer	42 (6.7)	52 (8.0)
Insurance type		
Self-pay	264 (42.2)	273 (42.1)
Essential plan^b^	48 (7.7)	60 (9.2)
Medicaid	153 (24.4)	170 (26.2)
Medicare	114 (18.2)	101 (15.6)
Other^c^	47 (7.5)	45 (6.9)
Language		
English	191 (30.5)	188 (29.0)
Chinese	63 (10.1)	54 (8.3)
Spanish	372 (59.4)	407 (62.7)
Age group, y		
18-44	17 (2.7)	13 (2.0)
45-54	288 (46.0)	313 (48.2)
55-64	180 (28.8)	192 (29.6)
≥65	141 (22.5)	131 (20.2)
Time since last portal login, d		
Never	100 (16.0)	100 (15.4)
0-7	197 (31.5)	191 (29.4)
8-21	104 (16.6)	105 (16.2)
22-90	111 (17.7)	119 (18.3)
≥90	114 (18.2)	134 (20.6)

^a^ Other race or ethnicity includes non-Hispanic American Indian or Alaska Native, Native Hawaiian or Other Pacific Islander, Middle Eastern or North African, multiracial, and any race or ethnicity not otherwise specified.

^b^ Essential plan refers to a low-cost New York State Marketplace health insurance program available to low-income individuals who are not eligible for Medicaid.

^c^ Other insurance includes commercial plans, grant-funded coverage, and cancer screening assistance programs.

Of 457 call group participants eligible for reminders (excluding patients who returned their FIT before day 7), nurses successfully reached 209 (45.7%), left voicemails for 67 (14.7%), and made unsuccessful attempts to contact 20 (4.4%). No call data were available for 161 participants (35.2%). As intended, no call attempts were documented in the text group. Among participants assigned to the text group, 94.5% of messages (1452 of 1537 messages) were successfully delivered.

### Primary Outcome

**Figure 2.**
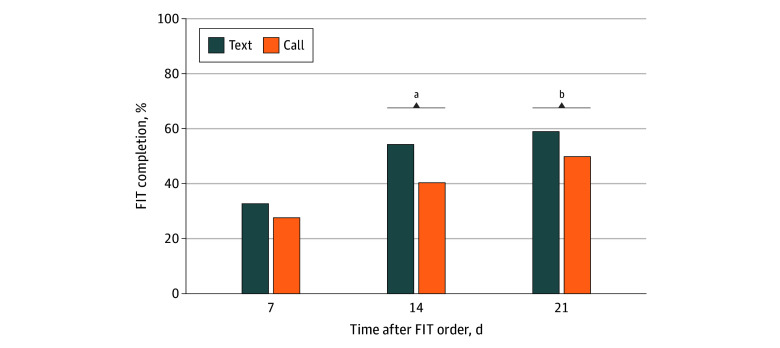
Bar Graph of Fecal Immunochemical Test (FIT) Completion Rates by Outreach Modality and Time Point ^a^*P* < .001. ^b^*P* < .025.

FIT completion rates were consistently higher in the text group across all follow-up intervals, with statistically significant differences at days 14 and 21 (Figure 2). At day 7, 212 of 649 participants (32.7%) in the text group had completed FIT screening compared with 173 of 626 (27.6%) in the call group (absolute difference, 5.0 percentage points; 95% CI, −0.0 to 10.1 percentage points; *P* = .051). By day 14, completion increased to 352 of 649 participants (54.2%) in the text group vs 252 of 626 participants (40.3%) in the call group (absolute difference, 14.0 percentage points; 95% CI, 8.6 to 19.4 percentage points; *P* < .001). At day 21, 382 of 649 participants (58.9%) in the text group had completed screening compared with 312 of 626 participants (49.8%) in the call group (absolute difference, 9.0 percentage points; 95% CI, 3.6 to 14.5 percentage points; *P* = .001). In multivariable logistic regression, assignment to the text group was associated with higher odds of FIT completion at 21 days (odds ratio, 1.58; 95% CI, 1.25 to 2.00; *P* < .001), corresponding to a 10.4–percentage point AME (95% CI, 5.2 to 15.7 percentage points). Full regression results are available in the eTable in Supplement 2.

### Exploratory Subgroup Analyses

Only the age interaction term was statistically significant. Those aged 18 to 44 years had the lowest odds of returning the FIT vs those older than 64 years (Table 3).

**Table 3.** ORs From Logistic Regression Models Assessing Effect Modification of Text Outreach on Fecal Immunochemical Test Return at 21 Days

Interaction model and subgroup variable	OR (95% CI)	*P* value
Treatment by race and ethnicity		
Asian	1.68 (0.55-5.05)	.29
Black	0.91 (0.33-2.51)	
Hispanic, Latino or Spanish	1.52 (0.74-3.16)	
White	1 [Reference]	
Other^a^	0.64 (0.23-1.79)	
Treatment by age group, y		
18-44	0.12 (0.01-0.96)	.007
45-54	1.77 (0.95-3.25)	
55-64	0.81 (0.41-1.60)	
≥65	1 [Reference]	
Treatment by payer		
Essential plan	0.46 (0.18-1.16)	.10
Medicaid	1.00 (0.56-1.80)	.99
Medicare	0.59 (0.30-1.16)	.13
Other	0.53 (0.20-1.38)	.20
Self-pay	1 [Reference]	NA
Treatment by sex		
Female	0.94 (0.58-1.54)	.82
Male	1 [Reference]	
Treatment by time since last portal login, d		
Never	1 [Reference]	
0-7	1.04 (0.50-2.18)	.59
8-21	1.73 (0.76-3.97)	
22-90	1.08 (0.48-2.44)	
≥90	0.99 (0.44-2.20)	

Abbreviations: NA, not applicable; OR, odds ratio.

^a^ Other race or ethnicity includes non-Hispanic American Indian or Alaska Native, Native Hawaiian or Other Pacific Islander, Middle Eastern or North African, multiracial, and any race or ethnicity not otherwise specified.

### Estimated Operational and Clinical Impact

Replacing telephone calls with text messages for FIT outreach offers measurable clinical and operational benefits. Given the call volume and observed effect, we estimated savings of 18 staff hours per month, return of 48 additional completed FITs within 21 days per month, of which an average of 2.2 would be abnormal, and detection of 1 to 2 additional CRC cases per year.

## Discussion

In this quality improvement randomized trial embedded within a large FQHC network, a behaviorally informed, automated text message strategy was associated with a 9.0–percentage point increase in FIT completion by 21 days compared with standard telephone outreach. Although telephone calls are commonly used for preventive care reminders, our findings suggest that automated, but thoughtfully designed, messaging can outperform live outreach in a clinical safety-net setting.

Text messaging likely succeeded through greater reach and, potentially, through text message content. Many patients in the call group were never reached, reflecting the practical limitations of manual outreach in busy clinical settings. In contrast, text messages can be delivered automatically and consistently. Although live communication may be more motivating under ideal conditions,^25^ our findings illustrate a trade-off between effectiveness and reliable delivery in clinical practice. Moreover, patients in the text group could receive up to 3 reminders, compared with a single telephone call in the control group, further amplifying differences in reach. More resource-intensive interventions, such as mailed FIT kits, patient navigation, or education, can yield larger effects, but these require substantial investment.^13,26^ Our results demonstrate that even a simple, automated reminder can still meaningfully boost screening uptake when more intensive efforts are not feasible.

Other randomized studies of text messaging for CRC screening have reported similar improvements, although most assessed longer follow-up intervals. In a trial among veterans mailed FIT kits, automated text reminders increased completion from 28% to 38% at 90 days (an increase of 10 percentage points).^27^ In a Spain-based study, text reminders 14 days after FIT pick-up increased return rates from 53.7% to 64.2% at 30 days (an increase of 10.5 percentage points).^28^ Our study adds evidence that effects can be observed earlier, within 3 weeks of the test order.

Behavioral design features may also have contributed to this program’s effectiveness. We incorporated several evidence-based nudges aimed at addressing common barriers to completing screening.^14^ One was the use of 3 separate reminder messages. Prior research on preventive care has shown that multiple reminders are more effective than a single reminder. Huf et al^24^ tested a bundled intervention including a prealert text, opt-out mailed FIT kit, and 3 follow-up text reminders, achieving a 17.7–percentage point higher return rate compared with only a single reminder. In vaccination, a megastudy of 19 influenza vaccine reminder strategies found that multimessage approaches outperformed single reminders, with the best 2-message strategy increasing uptake by 2.9 percentage points.^29^ When implementing multimessage strategies, however, it is important to note that repeated outreach can lead to message fatigue, highlighting the need to balance message frequency and efficiency.^30^ The messages also included a return-by deadline, which prior studies suggest can support planning and follow-through.^31,32,33^ Robb et al,^21^ for example, found that adding a 2-week suggested deadline to mailed FITs increased returns by 2 percentage points.

We also incorporated gain-framed wording (“this test could save your life”) and a social norms cue (“your provider is waiting”). Prior research shows that these types of message features can influence behavior, but effects are generally small and variable across studies.^34,35^ In CRC specifically, Hagoel et al^36^ reported that interrogative framing increased screening completion by 3 to 6 percentage points, and Muller et al^37^ found that culturally tailored messages improved colonoscopy uptake by 3.3 percentage points. Practitioner-linked cues have also proven effective: in a megastudy of influenza vaccination reminders, messages describing a shot as “reserved for you” increased uptake by 4.6 percentage points.^38^ Taken together, these findings suggest that while the impact of any single wording change may be small and inconsistent, such cues can serve as useful additions to broader outreach strategies.

Importantly, this study was not designed to disentangle the independent contributions of message content, delivery modality, and contact frequency. Isolating content effects would require an attention control, such as outreach matched in modality and frequency, which was beyond the scope of this study but may be investigated in a future study. Emerging automation technologies may also shift the trade-offs observed in this study. For example, artificial intelligence–assisted telephone outreach could enable repeated, standardized contact by telephone without increasing staff burden. Whether such approaches can achieve engagement comparable to that of text messaging, particularly as many patients might not answer calls from unknown numbers, remains an open question.

In our exploratory subgroup analysis, we found no statistically significant effect modification by race, ethnicity, insurance status, sex, or patient portal engagement. However, the effect of the intervention did appear smaller among participants aged 18 to 44 years. Although this suggests that this age bracket may be less responsive to text-based outreach, perhaps because screening recommendations were only recently extended to those younger than 50 years, further research is needed to confirm this finding and determine whether age-tailored messaging could enhance impact. The lack of differential effects by patient portal use also suggests that text message reminders can benefit patients regardless of prior digital engagement. We can also infer that older adults and publicly insured patients, groups often perceived as less digitally engaged, can still meaningfully benefit from thoughtfully designed digital outreach.^39,40^ Importantly, many subgroups had relatively small sample sizes, and the study may have been underpowered to detect modest differences in effect across groups.

Methodologically, this project demonstrates the feasibility of embedding rapid randomized evaluations into routine care. By integrating randomization into clinic workflows and using EHR data for outcome tracking, we were able to conduct a low-cost, minimally disruptive study that produced timely, actionable insights for clinic leadership. This rapid-cycle approach, adapted from A/B testing models in the technology industry, offers a scalable model for other health systems seeking to test and improve outreach strategies in clinical practice settings.^41^

### Limitations

This study has limitations that should be mentioned. This intervention was implemented across 1 clinic network in 1 geographic area and so these findings may not be generalizable to other contexts, although the methods used may be applicable elsewhere. Second, although behavioral strategies can mitigate cognitive barriers to screening, they do not address structural obstacles, such as unstable housing, transportation barriers, or competing priorities, that disproportionately affect FQHC populations and may persistently limit screening participation.

## Conclusions

In this quality improvement randomized clinical trial, a behaviorally informed, automated text message strategy improved FIT completion in a diverse primary care setting. Although simple and low-cost, the intervention produced clinically meaningful improvements in preventive care uptake with minimal operational burden. On the basis of these findings, the text message intervention was adopted across the FQHC network for all eligible patients with new FIT orders. Future work should focus on optimizing message content and frequency to prevent message fatigue, identifying which behavioral components are most effective, and ensuring these strategies remain impactful across diverse patient populations and settings.

## Notes

Supplement 1. Trial Protocol

Supplement 2. eTable. Complete multivariable logistic regression results with average marginal effects for FIT completion at 21 days

Supplement 3. Data Sharing Statement
