# Supplementary material for: Behaviorally Informed Text Messaging to Promote Colon Cancer Screening: A Quality Improvement Randomized Clinical Trial
Source: JAMA Netw Open. 2026 Apr 23;9(4):e267122. doi: 10.1001/jamanetworkopen.2026.7122 (PMC13107227; doi:10.1001/jamanetworkopen.2026.7122)
Supplement: Supplement 1. — Trial Protocol [file jamanetwopen-e267122-s001.pdf]

Rapid RCT Lab: Text messaging for colorectal cancer screeni

CIINICALTRIALS. GOV	Clinicaltrials.gov status	Registered
	Trial Registration Identifier	NCT06632054

INTRODUCTION	Background*	Colorectal cancer (CRC) screening reduces colorectal cancer incidence and mortality, but screening rates remain below national targets, particularly among underserved populations. Recent guideline changes lowering the recommended screening age to 45 years have also expanded the eligible screening population. Among eligible patients at the Family Health Centers at NYU Langone (FHCs), 59% were up-to-date with CRC screening in 2024.<br><br>At the FHCs, providers are prompted to order fecal immunochemical testing (FIT) annually for eligible patients aged 45 years and older. FIT kits are provided at in-person visits or mailed after virtual visits at no cost to the patient. Nurses provide education on FIT completion and currently conduct reminder phone outreach approximately 7 days after kit distribution for patients who have not yet completed the test. Despite this workflow, screening completion rates remain suboptimal.
	Problem analysis	FIT completion rates remain suboptimal despite FIT distribution during clinic visits and follow-up reminder calls. Current outreach relies on nurse-led phone calls, which require substantial staff time and may fail to reach a large proportion of patients.
	Baseline data*	667 FIT orders placed in February 2024
	Observations	Among FIT orders placed in February 2024, 424/667 (63%) remained unresulted after 1 week.
	Objective(s)	To increase rates of FIT completion among eligible patients at the FHCs
	Project location	Family Health Centers at NYU Langone in Brooklyn, New York. Patients enrolled in the Community Medicine Program will be excluded due to a concurrent CRC screening study.
	Planned end study date	Jul-25
	Key stakeholders	Family Health Centers clinical leadership
	Project lead	Arielle Elmaleh-Sachs/ arielle.elmaleh-sachs@nyulangone.org
	Team members	Olivia Korostoff-Larsson; William King; Elan Pelegri; Doreen Colella; Isaac Dapkins; Kelly Eng; Nathan Klapheke; Holly Krelle; Nick Mahieu; Erika McManus; George Shahin; Arielle Elmaleh-Sachs; Leora Horwitz

INTERVENTIONS	Intervention A vs. Intervention B description	Intervention A: Automated text reminders sent on days 2, 5, and 8 after FIT order placement. Messages include behavioral nudges: repetition, deadline, gain framing, and personalization.<br><br>Intervention B (current practice): Standard nurse-led outreach consisting of a reminder phone call 8 days after FIT order placement with voicemail and one repeat attempt within 72 hours if no answer.

OUTCOMES	Primary outcome	FIT testing completion within 21 days of FIT test order
	Rationale	Timely return and processing of FIT kits is required for screening completion. A 21-day outcome allows sufficient time for patients to realistically complete and return the test while focusing on early follow-through, when reminder outreach is most likely to influence behavior. Evaluating completion within this timeframe also supports rapid-cycle testing of outreach strategies and potential iteration of messaging interventions.
	Is this outcome currently routinely captured in clinical care	Yes (routinely captured in EHR)
	Baseline performance	Baseline completion rates as of February 2024:<br>- After 7 days: 32.9%<br>- After 14 days: 46.0%<br>- After 21 days: 51.7%
	Minimum clinically important effect size	10 percentage point increase in FIT completion
	Secondary outcome(s)	FIT testing completion within 7 and 14 days of FIT test order
	Balancing outcome(s)	Monitoring for patient complaints or opt-outs from text messaging
	Subanalyses	Exploratory subgroup analyses will be conducted using logistic regression models to assess whether the effect of the text messaging intervention varies by patient characteristics.
	Demographic characteristics	Race/ethnicity, sex, age, insurance type, days since last MyChart login, language, clinic site (all routinely captured in the EHR)
	Other cohorts, if any	N/A
	Unintentional consequences	Possible message fatigue leading to reduced responsiveness to this or other messaging interventions; dissatisfaction with automated outreach if perceived as a replacement for live communication; or increased patient requests to opt out of text messaging
	Conditions for continuing/terminating the project	The intervention will be evaluated after completion of the planned study period to determine whether the text messaging strategy should be implemented in routine outreach workflows. Significant disruptions to clinic operations or concerns raised by Family Health Centers leadership may prompt modification or early discontinuation of the study.
	Specify factors to consider if no significant difference found for the primary outcome (e.g., any improvement in primary outcome, secondary outcomes).	If no statistically significant difference is observed in FIT completion between groups, additional considerations will include any directional improvement in the primary or secondary outcomes, and operational implications of the intervention. In particular, the study team will assess potential reductions in nursing staff time required for reminder outreach and feedback from clinic staff regarding workflow efficiency and acceptability of automated text messaging compared with phone-based outreach.

TS	Study population definition	Adult patients (age ≥18 years) with a new FIT order placed at any Family Health Centers at NYU Langone site during the study period
	Exclusion criteria	Patients will be excluded if they: are younger than 18 years of age; have previously opted out of institutional text messaging; have a preferred language other than English, Spanish, or Chinese; are enrolled in the Community Medicine Program due to a concurrent CRC screening initiative
	Expect N/week	Expected 167 FIT orders per week based on 2024 data.

PARTICIPAN	N required to reach desired effect size with 80% power	7 day completion: 738 (4.8 weeks)<br>14 day completion: 784 (5.1 weeks)<br>21 day completion: 738 (4.8 weeks)

RANDOMIZATION	**Unit of randomization (patient, provider, hospital-level)**	Patient
	**Allocation ratio**	1:1
	**Sequence generation**	
	**Method for generating the random allocation sequence**	Randomize through software (R)
	**Type of randomization, details of any restriction (e.g., blocking & blocking size)**	Simple randomization
	**Allocation concealment mechanism**	
	**Mechanism for implementing random allocation sequence**	Patients assigned random number between 0 and 1, <=0.5 assigned to control and >0.5 assigned to intervention.
	**Implementation**	
	**Who will generate random allocation sequence**	Data analyst
	**Who will enroll participants**	Data analyst, using inclusion/exclusion criteria
	**Who will assign participants to interventions**	Data analyst
	**Blinding**	
	**Patient? Y/N**	Not possible
	**Provider? Y/N**	N
	**Investigator? Y/N**	N
	**Data Analyst? Y/N**	N

DATA ANALYSIS	**Analytical approach**	The primary and secondary outcomes will be compared between groups using chi-square tests.<br><br>Exploratory multivariable logistic regression analyses will be conducted to assess the association between intervention assignment and FIT completion while adjusting for patient characteristics and to explore heterogeneity of treatment effect across prespecified subgroups.
	**Rationale**	The primary outcome is binary and treatment assignment is randomized; therefore, unadjusted comparisons between groups are appropriate for the primary analysis.<br><br>Logistic regression will be used for exploratory adjusted analyses and subgroup analyses.

*Template based on 2017 CONSORT Checklist of Information to Include When Reporting Randomized Trials Assessing NPTS.
