# Supplementary material for: Behaviorally Informed Text Messaging to Promote Colon Cancer Screening: A Quality Improvement Randomized Clinical Trial
Source: JAMA Netw Open. 2026 Apr 23;9(4):e267122. doi: 10.1001/jamanetworkopen.2026.7122 (PMC13107227; doi:10.1001/jamanetworkopen.2026.7122)
Supplement: Supplement 2. — eTable. Complete multivariable logistic regression results with average marginal effects for FIT completion at 21 days [file jamanetwopen-e267122-s002.pdf]

Supplemental Online Content

Korostoff-Larsson O, King W, Pelegri E, et al. Behaviorally informed text messaging to promote colon cancer screening: a quality improvement randomized clinical trial. *JAMA Netw Open*. 2026;9(4):e267122. doi:10.1001/jamanetworkopen.2026.7122

eTable. Complete multivariable logistic regression results with average marginal effects for FIT completion at 21 days

This supplemental material has been provided by the authors to give readers additional information about their work.

© 2026 Korostoff-Larsson O et al. *JAMA Network Open*.

eTable. Complete multivariable logistic regression results with average marginal effects for FIT completion at 21 days

Variable	Odds Ratio (95% CI)	Average Marginal Effect (95% CI)	p-value
**TREATMENT**			
Text	1.58 (1.25, 2.00)	+10.4 pp (5.2, 15.7)	<0.001
Call (ref)	—	—	—
**RACE / ETHNICITY**			
Asian	8.49 (4.89,15.20)	+43.0 pp (33.5, 52.5)	<0.001
Black or African American	2.19 (1.33, 3.63)	+18.6 pp (7.0, 30.3)	0.002
Hispanic or Latino or Spanish	1.43 (0.99, 2.07)	+8.5 pp (–0.2, 17.3)	0.06
Other	1.25 (0.75, 2.08)	+5.3 pp (–6.9, 17.4)	0.39
Prefer not to answer	1.15 (0.68, 1.95)	+3.4 pp (–9.1, 15.9)	0.59
White (ref)	—	—	—
**AGE**			
45–54	0.43 (0.18, 0.97)	–18.4 pp (–35.4, –1.4)	0.03
55–64	0.55 (0.23, 1.24)	–13.1 pp (–30.4, 4.2)	0.14
65+	0.96 (0.38, 2.33)	–0.8 pp (–19.3, 17.8)	0.94
18-44 (ref)	—	—	—
**INSURANCE TYPE**			
Essential Plan	0.95 (0.60, 1.51)	–1.2 pp (–11.4, 9.1)	0.83
Medicaid	0.58 (0.43, 0.79)	–12.1 pp (–18.9, –5.3)	<0.001
Medicare	0.47 (0.29, 0.75)	–17.1 pp (–27.3, –6.9)	0.001
Other	0.79 (0.48, 1.30)	–5.3 pp (–16.5, 5.9)	0.35
Commercial (ref)	—	—	—
**SEX**			
Female	0.91 (0.71, 1.16)	–2.2 pp (–7.7, 3.4)	0.44
Male (ref)	—	—	—
**DAYS SINCE LAST PORTAL LOGIN**			
0–7 days	1.08 (0.74, 1.58)	+1.8 pp (–6.8, 10.4)	0.68
8–21 days	1.21 (0.79, 1.86)	+4.4 pp (–5.2, 13.9)	0.37
22–90 days	1.05 (0.70, 1.60)	+1.2 pp (–8.3, 10.7)	0.80
90+ days	0.92 (0.61, 1.37)	–2.0 pp (–11.2, 7.2)	0.68
Never (ref)	—	—	—

© 2026 Korostoff-Larsson O et al. *JAMA Network Open*.
