# Supplementary material for: Behaviorally Informed Text Messaging to Promote Colon Cancer Screening: A Quality Improvement Randomized Clinical Trial
Source: JAMA Netw Open. 2026 Apr 23;9(4):e267122. doi: 10.1001/jamanetworkopen.2026.7122 (PMC13107227; doi:10.1001/jamanetworkopen.2026.7122)
Supplement: Supplement 3. — Data Sharing Statement [file jamanetwopen-e267122-s003.pdf]

Data Sharing Statement

Korostoff-Larsson. Behaviorally Informed Text Messaging to Promote Colon Cancer Screening. *JAMA Netw Open*. Published April 23, 2026. doi:10.1001/jamanetworkopen.2026.7122

Data
Data available: Yes
Data types: Deidentified participant data
How to access data: Deidentified data available on request to the corresponding author at leora.horwitz@nyulangone.org
When available: With publication

Supporting Documents
Document types: None

Additional Information
Who can access the data: Anyone requesting the data
Types of analyses: For any purpose
Mechanisms of data availability: On approval of a proposal by the corresponding author
